# Increased Diacylglycerols Characterize Hepatic Lipid Changes in Progression of Human Nonalcoholic Fatty Liver Disease; Comparison to a Murine Model

**DOI:** 10.1371/journal.pone.0022775

**Published:** 2011-08-09

**Authors:** D. Lee Gorden, Pavlina T. Ivanova, David S. Myers, J. Oliver McIntyre, Michael N. VanSaun, J. Kelly Wright, Lynn M. Matrisian, H. Alex Brown

**Affiliations:** 1 Department of Cancer Biology and Vanderbilt Ingram Cancer Center, Vanderbilt University Medical Center, Nashville, Tennessee, United States of America; 2 Department of Pharmacology, Vanderbilt University Medical Center, Nashville, Tennessee, United States of America; 3 Department of Surgery, Vanderbilt University Medical Center, Nashville, Tennessee, United States of America; 4 The Vanderbilt Institute of Chemical Biology, Vanderbilt University Medical Center, Nashville, Tennessee, United States of America; University of South Alabama, United States of America

## Abstract

**Background and Aims:**

The spectrum of nonalcoholic fatty liver disease (NAFLD) includes steatosis, nonalcoholic steatohepatitis (NASH), and progression to cirrhosis. While differences in liver lipids between disease states have been reported, precise composition of phospholipids and diacylglycerols (DAG) at a lipid species level has not been previously described. The goal of this study was to characterize changes in lipid species through progression of human NAFLD using advanced lipidomic technology and compare this with a murine model of early and advanced NAFLD.

**Methods:**

Utilizing mass spectrometry lipidomics, over 250 phospholipid and diacylglycerol species (DAGs) were identified in normal and diseased human and murine liver extracts.

**Results:**

Significant differences between phospholipid composition of normal and diseased livers were demonstrated, notably among DAG species, consistent with previous reports that DAG transferases are involved in the progression of NAFLD and liver fibrosis. In addition, a novel phospholipid species (ether linked phosphatidylinositol) was identified in human cirrhotic liver extracts.

**Conclusions:**

Using parallel lipidomics analysis of murine and human liver tissues it was determined that mice maintained on a high-fat diet provide a reproducible model of NAFLD in regards to specificity of lipid species in the liver. These studies demonstrated that novel lipid species may serve as markers of advanced liver disease and importantly, marked increases in DAG species are a hallmark of NAFLD. Elevated DAGs may contribute to altered triglyceride, phosphatidylcholine (PC), and phosphatidylethanolamine (PE) levels characteristic of the disease and specific DAG species might be important lipid signaling molecules in the progression of NAFLD.

## Introduction

Nonalcoholic fatty liver disease (NAFLD), characterized by steatosis and progression to steatohepatitis (NASH) and cirrhosis, is a problem of increasing clinical significance. Statistics in the United States suggests that 31% of the adult population have hepatic steatosis [1]. Additionally, obesity is associated with a number of malignancies including breast and colon carcinomas [2]. Currently, NAFLD can only be definitively diagnosed by liver biopsy, but it is suspected in patients with obesity and/or syndromes of insulin resistance accompanied by elevated serum aminotransferase levels, commonly referred to as the metabolic syndrome [3], [4].

The detailed composition of the lipids accumulating in NAFLD is not well characterized on a species level. Lipidomics technologies permit characterization of lipids in biological systems at a level previously not possible using traditional methods such as thin-layer chromatography (TLC), gas chromatography (GC), or high performance liquid chromatography (HPLC) alone. Prior reports about the phospholipid distribution in NAFLD have also shown the importance of the phosphatidylcholine (PC)/phosphatidylethanolamine (PE) ratio on liver disease progression from steatosis to steatohepatitis and its implication as a key regulator of cell membrane integrity [5]. However, the phospholipid composition and the redistribution of the increased fatty acids within specific phospholipid classes as well as important diacylglycerol (DAG) changes have not been adequately investigated. Such information has more often been studied at the level of total DAG or by gas chromatography to provide fatty acid specific data rather than at a lipid species level [6]. Changes in DAG ratios and species may be important in lipid signaling and in the progression of NAFLD.

Multiple animal models have been developed to address metabolism, signaling pathways, and genetic variance that affect the development of steatosis and steatohepatitis [7], [8]. Genetically modified mice bred for studies in obesity have also been investigated by mass spectrometry (MS) for species level changes of DAGs, TAGs, and some phospholipids [9]. Steatosis induced by high-fat diet is a relevant murine model that we now show reflects the spectrum of lipid changes associated with human NAFLD.

Application of mass spectrometry techniques in conjunction with computer assisted analysis [10] has allowed determination of specific phospholipid and DAG species from liver biopsies and comparison during disease progression. Defining patterns of specific lipid species across the spectrum of NAFLD may prove clinically useful in the early diagnosis and identification of patients with this disease. Improved insights into lipid signaling pathways important in progression of NAFLD may also be valuable for understanding molecular mechanisms involved in disease progression.

## Methods

### Ethics Statement

Mice were housed in the accredited animal facility at Vanderbilt University. Studies were approved by the Vanderbilt Animal Care and Use Committee (protocol # M/07/262) and every effort to minimize pain and suffering to the animals was made. Human tissues were collected under a protocol approved by Vanderbilt's Internal Review Board (IRB#: 061257). Biopsies for research analysis were obtained from clinical biopsy specimens in all cases and did not require separate consent.

### Murine

Eight week old C57bl/6J male mice from Jackson Research Laboratories were fed a 13.5% fat “control” diet (5001, LabDiet: 13.5% calories from fat, 58% from carbohydrates, and 28.5% from protein) or a 42% fat “Western” diet (TD.88137, Harlan Teklad: 42% calories from fat, 42.7% from carbohydrates, and 15.2% from protein) ad libitum for 12 weeks.

### Human

Normal human samples were obtained from donor liver biopsies for liver transplantation. Cirrhotic specimens were recipient livers during liver transplant. Clinical etiology of cirrhosis was NASH. Steatotic specimens were obtained from clinical biopsies taken from morbidly obese patients undergoing gastric bypass surgery. Patients had no history of ethanol induced steatosis. Three steatotic samples analyzed had <10% steatosis on histologic examination. In spite of mild steatosis in these, the differences described in the three patient groups remain robust. Specimens were immediately taken from the operating room and processed for lipidomics analysis without frozen storage. A portion of the human samples were used only for phospholipid identification by tandem mass spectrometry in each condition, while the remainder were used for global lipid profiling and DAG analysis.

### Preparation and Quantitation of Histologic Specimens

Human and murine liver tissue sections were stained with Hematoxylin for 5 minutes followed by Eosin counterstaining for 1 minute. Quantitation of percent steatosis in human biopsy specimens was performed in a blinded fashion by pathologists specializing in liver pathology at Vanderbilt University Medical Center.

Additionally, Oil red O staining was done using freshly isolated human and murine liver tissue which was fixed in 10% neutral buffered formalin at 4°C for two days, transferred to a 20% sucrose solution for two days and then frozen in Tissue-Tek. Frozen tissues were sectioned at 8 um on a Microm cryostat set to −19°C and then air dried. In brief, rehydrated liver sections were stained with Oil red O from 5% stock solution in isopropanol diluted at 3∶2 with distilled water for 20 minutes and counterstained with Mayer's Hematoxylin as previously described [14].

### Glycerophospholipid Analysis

Liver biopsies (10–20 mg) were immediately frozen in liquid nitrogen and phospholipids were extracted as described previously [11]. Briefly, the frozen samples were placed in a tight-fit glass homogenizer (Kimble/Kontes Glass Co., Vineland, NJ), 800 µl of cold 0.1 N HCl:CH_3_OH (1∶1) was added, and the sample was homogenized for about 1 min on ice. The suspension was transferred to a cold 1.5 ml microfuge tube, 400 µl of ice-cold CHCl_3_ was added and the extraction proceeded with vortexing (1 min) and centrifugation (5 min, 4°C, 18, 000 xg) to separate the layers. The lower organic phase was collected in a new cold microfuge tube and solvent evaporated under vacuum (Labconco Centrivap Concentrator, Kansas City, MO). Samples were redissolved in 80 µl of CH_3_OH:CHCl_3_ (9∶1) and 1 µl of NH_4_OH (18 M) was added before analysis. Mass spectral analysis was performed on a Finnigan TSQ Quantum triple quadrupole mass spectrometer (ThermoFinnigan, San Jose, CA) equipped with a Harvard Apparatus pump and an electrospray source. Samples were analyzed at an infusion rate of 10 µl/min in both positive and negative ionization modes over the range of *m/z* 350–1200. Data were collected with the Xcalibur software package (ThermoFinnigan) and analyzed with software developed in our laboratory. Identification of individual glycerophospholipids was accomplished by tandem mass spectrometry (ESI-MS/MS) and was based on their chromatographic and mass spectral characteristics [12], [15]. This analysis allows identification of the two fatty acid moieties but does not determine their position on the glycerol backbone (*sn-1* vs. *sn-2*). Therefore, glycerophospholipid species are referred to throughout using X:Y notation (X-total number of carbons, Y-total number of double bonds in both FA, e.g., PE 36∶1).

### Diacylglycerol Analysis

Frozen samples were homogenized as for the glycerophospholipid analysis and after extraction, diacylglycerols (DAGs) were separated from more polar phospholipids using a silica gel column chromatography using isocratic elution with 65∶35∶0.7 (v/v/v) CHCl_3_:CH_3_OH:H_2_O and analyzed by mass spectrometry as sodium adducts as described elsewhere [13].

### Data Analysis

Global glycerophospholipid analysis utilized three internal standards per ionization mode (positive for PCs, negative for all others). Following normalization, relative peak heights of species within classes were examined and tables of ratios of intensity within each class were prepared, similarly to Rouzer et al. [10] Three replicates were used for each biologically independent sample. Species-level statistical analysis was performed by ANOVA (human samples with three conditions) or Student's t test (murine samples with two conditions). Quantification and data analysis of DAGs was conducted similarly to Callender et al. [13]. Principal components analysis was carried out in MATLAB.

## Results

**Figure 1:**
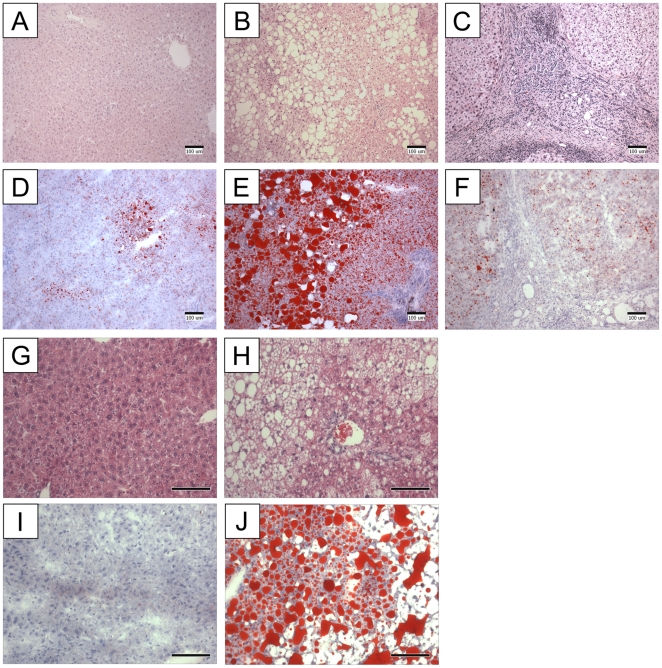
Panels A–F show histological progression of nonalchoholic fatty liver disease in human biopsies. (**A,B,C**) show hematoxylin and eosin (H&E) staining, and (**D,E,F**) show oil red O (ORO) staining. (**A,D**) normal; (**B,E**) steatohepatitis; (**C,F**) cirrhosis. In (**A**) normal lobular architecture of the liver is observed, with little fatty change on ORO staining in (**D**). Changes with progression to steatosis are seen with increased fat globules. The presence of inflammatory cell infiltrates (**B**) and lipid droplets are prominently visible in (**E**). Cirrhotic changes show characteristic fibrotic pattern (**C**) and a paucity of staining with ORO in (**F**). *Panels G*–*J show histology of normal murine liver specimens.* (G) H&E staining as well as oil red O staining (H). Fatty liver disease in murine tissues is histologically demonstrated by H&E (I) and using oil red O staining (J).

Clinical samples used for mass spectrometry analysis of human liver lipids revealed typical histology associated with NAFLD (**Figure 1** **panels A–F**). Murine samples demonstrated typical histology of fatty liver disease (**Figure 1** **panels G–J**). For lipidomic analysis, NAFLD samples were classified as steatotic. Steatotic specimens showed marked staining with ORO and various levels of inflammatory infiltrates associated with steatohepatitis. Cirrhotic specimens showed characteristic dense fibrosis and lacked abundant lipid. Similar patterns associated with steatosis were observed in the livers of mice fed a high fat diet [14].

Clinical characteristics of patients included in normal, steatotic, and cirrhotic groups are shown in Table 1.

**Table 1:** Clinical data for sample populations. Patient ages, body mass index (BMI) and serum liver function tests are represented as mean values followed by a range in parentheses.

	Normal (n = 12)	NAFLD (n = 17)	Cirrhosis (n = 9)
Female	2	15	2
BMI	23.6 (19.6–33.8)	59 (42.1–85.8)	
Age	39.6 (18–62)	40.3 (20–50)	57.8 (49–69)
Serum AST	47.1 (18–64)	30 (16–80)	62.3 (33–110)
Serum ALT	35.8 (18–53)	29 (16–84)	38.6 (16–71)
Histologic Grade Steatosis	<5–10%	10–80%	<5%
Histologic Grade Fibrosis	none	mild -moderate	severe

A mass spectrometry lipidomics approach was used to examine species-specific phospholipid and DAG profiles in human liver biopsies to assess changes associated with progression of NAFLD. Glycerophospholipid species were identified within the six major phospholipid classes (phosphatidylserine PS, phosphatidylinositol PI, phosphatidylethanolamine PE, phosphatidylcholine PC, phosphatidic acid PA, and phosphatidylglycerol PG).

### Human Data

**Figure 2:**
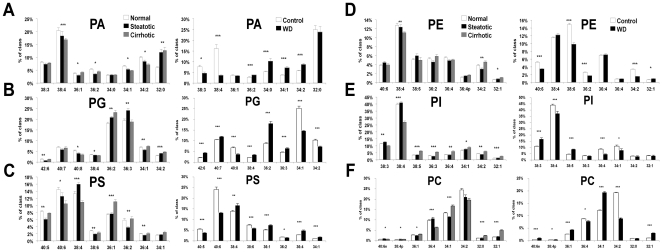
Selected glycerophospholipid species distribution from human and murine livers. (A, B, C, D, E, F) Data from humans **(**represented in graphs on left side of lettered panels)(black bars: normal; open: steatotic; hatched: cirrhotic) for phosphatidic acid (PA), phosphatidylglycerol (PG), phosphatidylserine (PS) classes, respectively; n = 5–9 individuals per group. Graphs on right side of each panel demonstrate results in mouse model (black bars: control; open: WD); n = 3 individual mice per group. Error bars = sem. Asterisks indicate Bonferroni adjusted significance at better than the *p<0.05, **p<0.01, ***p<0.001 levels from ANOVA across disease states for human data and from Student's t test for murine data.

To elucidate which lipids were altered in steatotic (NAFLD) and cirrhotic disease, species-level analysis of phospholipids from human liver biopsies were compared by ANOVA. This comparison revealed 59 species (out of 154 measured) with significant changes after applying a Bonferroni correction. Selected species are depicted in **Figure 2** **.** Species of PA changed similarly in steatotic and cirrhotic when compared to normal controls, with a clear increase in saturated and monounsaturated fatty acid (FA) containing PA species in both types of diseased liver samples. The changes in PG species were also similar in both steatotic and cirrhotic liver as compared with normal samples, with the greatest relative difference being an increase in 36∶2 PG (primarily consisting of 18∶1/18∶1 FA) (28% cirrhotic vs. normal). PS species showed a different pattern of changes in the two disease stages. Although several of the polyunsaturated FA (PUFA) containing PS species (e.g., 40∶4 – not shown, 40∶5, 40∶6) decreased similarly in both conditions, 38∶4 PS increased in steatotic but decreased in cirrhotic liver. Further, in cirrhotic samples there was an increase in PS species containing at least one monounsaturated FA, i.e., PS (36∶1, 36∶2, 34∶1- containing 18∶1 FA), while these species either decreased slightly or did not change in steatotic liver samples. As with PS, lipid species in the other three major lipid classes, PI, PE, and PC also showed changes that were distinct in steatotic and cirrhotic livers. While PUFA-containing PE and PC (e.g., 38∶4 – not shown, 36∶4) decreased in cirrhotic samples, saturated, mono-, and di-unsaturated FA containing PE and PC increased compared to normal (including PC 34∶0 and PE 34∶1 which also increase 30%, p<0.001 – data not shown). By contrast, in steatotic liver samples these same species either remained mostly unchanged or showed only small changes in the opposite direction. Interestingly, analysis of PI species did not show any significant differences between normal and steatotic liver samples. However, in the cirrhotic samples there was a decrease in the major PUFA-containing PI (38∶4) compared to normal liver while almost all of the other detected PI species were elevated in this diseased state compared to normal.

There were differences in PC plasmalogens between disease states, as PUFA species in the 34:X, 36:X, and 38:X series increased in the steatotic samples. The overall fraction of the PC subclass which contained plasmalogen species increased 28% in the steatotic cases over normal (p<0.001), but did not significantly increase in the cirrhotic cases as compared to normal. In contrast, while PE plasmalogens as a group showed less difference between steatotic and normal samples, several individual PE plasmalogen species increased more than 33% (e.g., PE 36:4p, 38:4p, 38:5p, all p<0.05) in the cirrhotic cases as compared to normal.

Overall, a similar number of glycerophospholipid species were identified in all three human sample types. The number of identified species differs from the number of species monitored in the MS analysis as some of them are isobaric or detected at trace levels. Samples from steatotic and cirrhotic livers appeared to have more plasmalogen PC species detected than normal (Table S1). Notably, almost twice as many PS species were identified in cirrhotic liver samples compared to the normal and steatotic conditions, which each had a similar number of PS species represented. One notable difference in cirrhotic livers was the finding of a rare lipid 38∶4 PIe (18:0e, 20∶4) which was only detected in cirrhotic liver samples. Since the presence of ether-PI in tissues has not been previously reported, the identification was confirmed by comparison of retention time and fragmentation spectra to a chemically defined synthetic standard (38:4e PI, 1-octadecyl-2-(5Z, 8Z, 11Z, 14Z-eicosatetraenoyl)-*sn*-glycero-3-phospho-(1′-myo-inositol), obtained from Avanti Polar Lipids) as described elsewhere [15]. (Figure S1)

In addition, analysis also revealed decreases in arachidonic acid-containing lipid species in the majority of phospholipid classes in human liver specimens at different disease stages, primarily in cirrhosis.

### Murine Data

Lipid species in livers of mice fed a control (CON) or Western diet (WD) showed significant changes in 102 (of 147 measured) lipid species (Bonferroni corrected Student's t-test). Results are shown for selected species in each class in **Figure 2**. Ratiomic changes were most evident in PC, PS, and PG species when comparing WD and control samples. Although there were similarities in how the murine WD/CON lipid profile and the steatotic/normal human lipid profiles diverged (e.g., particularly for PS), the mouse WD/CON contrasts bear even stronger resemblance to the way human cirrhotic livers differed in the steatotic/normal conditions (manifested mainly within the PS, PI, and PC classes). There were additional differences in human and murine profiles. Of note, one species of PI (36∶0) was found to decrease substantially in WD vs. CON samples, but was completely undetected in human liver samples. Concomitant with this change, LPI 18∶0 increased by 27% (p<0.001) with the high fat diet over the controls.

Certain lipids showed coherent changes in mice and humans. Similar to the human liver data, the percentage of the PC subclass which contained plasmalogen species increased 23% in the WD mice versus CON (p<0.001), but the fraction of PE plasmalogen species was unaffected by diet. The portion of the PC class signal arising from 20∶4 fatty acid-containing lipids was also unchanged in WD versus CON, but this percentage increased in other classes (PI+20%, PS+31%, both p<0.001) and decreased dramatically in the primary 20∶4 containing PA species, 38∶4 (−78%, p<0.001).

### Changes in Diacylglycerol Levels

**Figure 3:**
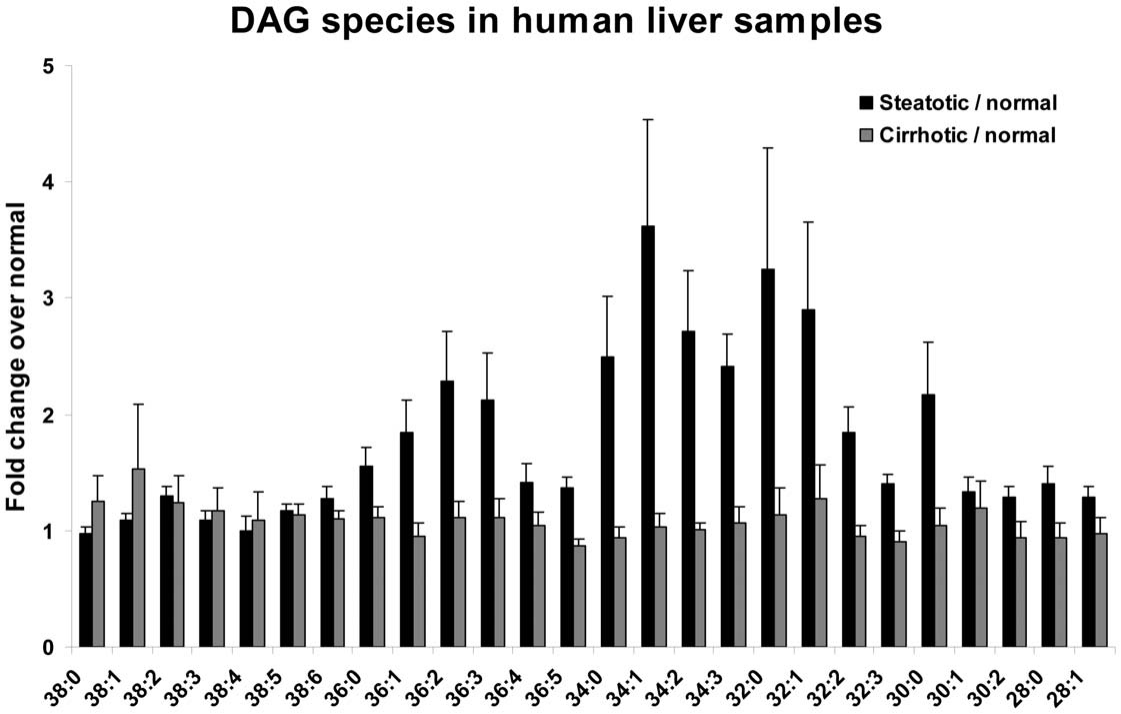
Fold changes in diacylglycerol (DAG) species from human livers. Fold changes in DAGs from human steatotic and cirrhotic samples versus normal samples. All fold changes for steatotic/normal samples in panel A are significant with p<0.05 except for 38∶0, 38∶1, 38∶3, 38∶4. No fold changes for cirrhotic/normal samples in panel A are significant except 38∶0,38∶1,38∶2 (p<0.0122); n = 5–9 individuals per group. Error bars = sem.

Species analysis of TAG precursors, diacylglycerols, demonstrated multiple fold increases in relatively short chain (30–36 carbon atoms) species containing 0, 1, 2, and 3 double bonds (**Figure 3** **,** **Figure 5** **)**. The increases were large in human steatotic livers compared to normal but minimally detectable in the cirrhotic livers. To ensure that the changes observed reflected strong differences between disease states, the DAG levels were compared by gender as well. No differences with p<0.01 were found between genders (data not shown).

**Figure 4:**
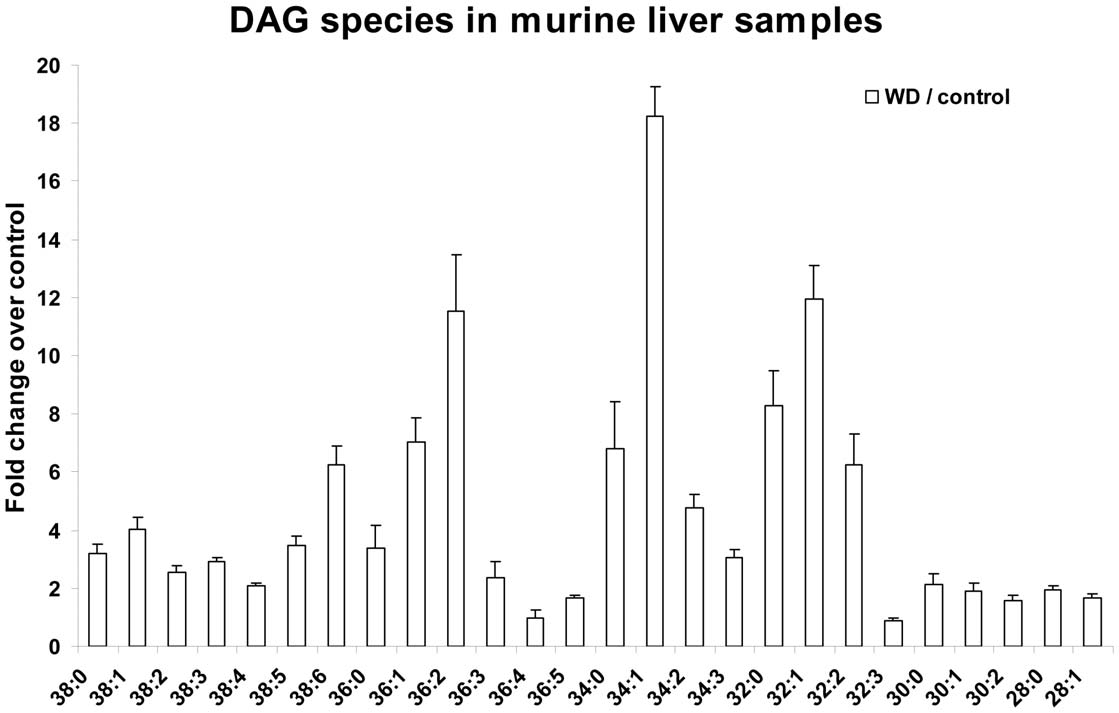
Fold changes in diacylglycerol (DAG) species from murine livers. Fold changes (WD/control) in DAGs from murine tissues. All fold changes in panel B are significant with p<0.024 except for 36∶4; n = 3 individuals per group.

Similar analysis of DAGs in murine livers showed large (>4 fold in ten molecular species) increases with the Western diet versus the controls. The pattern of DAG changes in the steatotic animals (WD) was similar to those seen in the human steatotic liver biopsies with the exception of the 38:X DAGs, which showed no change in human samples but a modest increase in the murine livers (**Figure 4** **)**.

**Figure 5:**
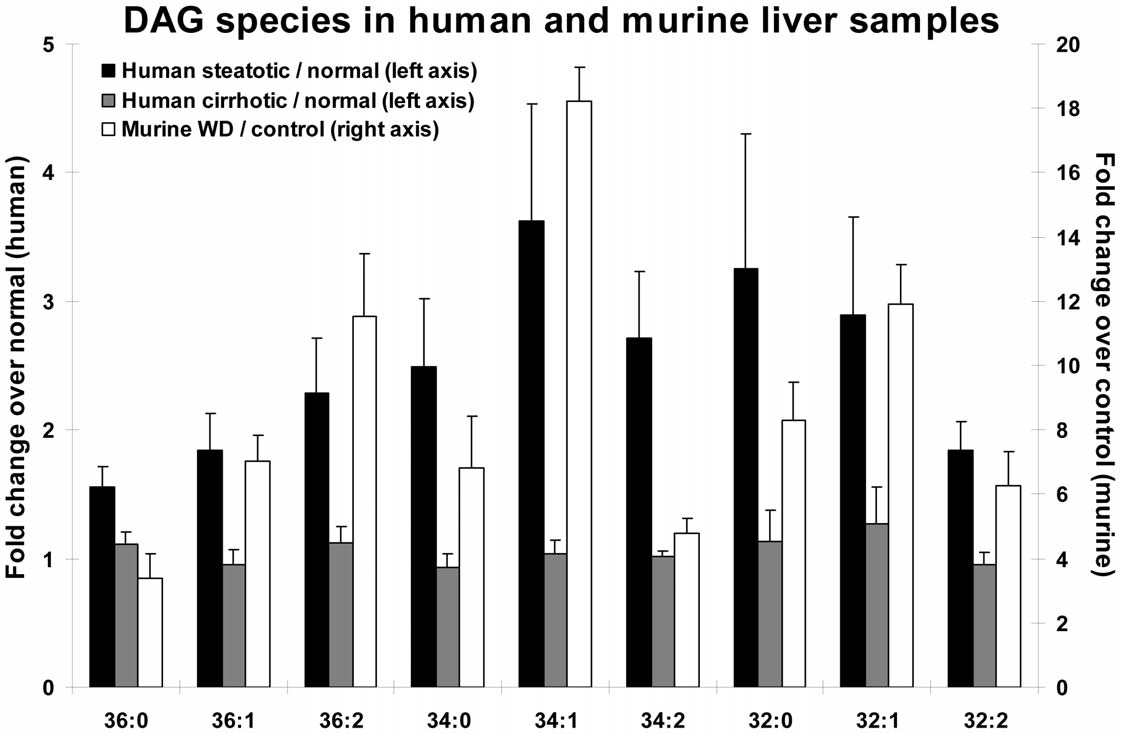
Fold changes in diacylglycerol (DAG) species from human and murine samples. Comparison between DAG fold changes in mouse and human liver samples (black bars: human steatotic/normal; gray bars: human cirrhotic/normal; white bars: murine WD/control). All fold changes for WD/control and steatotic/normal are significant with p<0.0105; none of the fold changes for cirrhotic/normal are significant at p<0.05.

Importantly, DAG species analysis in the murine samples closely resembled the changes in human steatotic livers, but demonstrated a greater magnitude. Again, the most dramatic differences were increases in monounsaturated FA–containing species (36∶2, 34∶1, 32∶1) in livers from mice fed a high-fat diet compared to controls.

### Principal Components Analysis (PCA) of Glycerophospholipids reveals distinct profiles by condition

**Figure 6:**
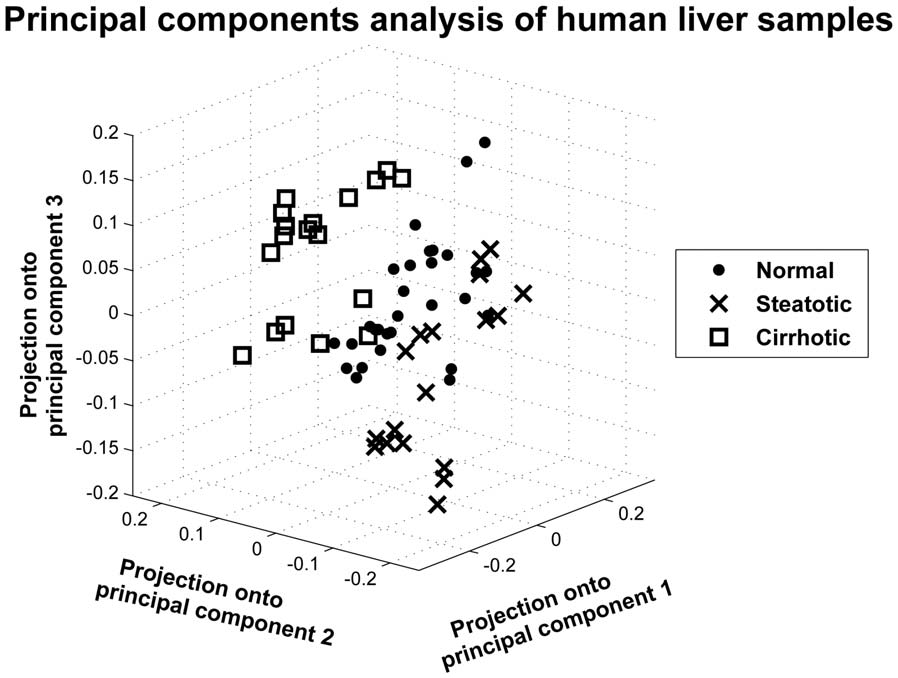
Principal component analysis of human lipidomics data. Human samples for different conditions: normal (dots), steatotic (crosses), cirrhotic (squares). All replicates are shown.

Compared to the DAGs, there were relatively smaller magnitude differences in the glycerophospholipids with the disease state (humans) or diet (mice). For a broad perspective, the glycerophospholipid profiles of the human liver and mouse liver were analyzed and compared using eigenanalysis. A plot of the first three principal eigenvectors of variability for the human data (**Figure 6**) showed that the profiles of these lipids inherently cluster within disease states (normal, steatotic, cirrhotic), validating that despite a paucity of changes on a lipid species basis (as with the DAG data), the global profiles between disease conditions were highly distinct. In particular, the second principal component efficiently separated the cirrhotic samples from the others based on their lipid profile, while the first and third principal components could be used to largely segregate the steatotic and normal samples. These three principal components together explain 58% of the variance in the human global lipid data. The ANOVA p-value comparing the three tissue sample types was significant for each of the first three principal components (p<0.001). Similar to the above analysis for DAG with respect to gender, the first two principal components showed no difference between males and females, while the third leading component was significant when comparing genders (p<0.01) but with considerably less contrast than across disease states (p<1×10^−5^).

**Figure 7:**
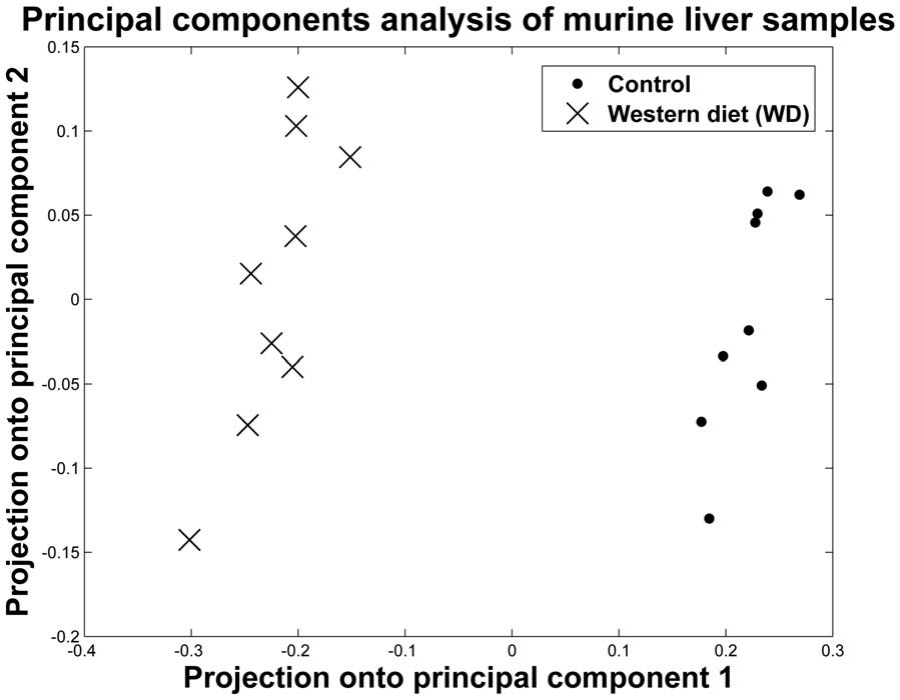
Principal component analysis of murine lipidomics data. Murine samples for different diets (control, dots; Western diet = WD, crosses). All replicates are shown.

PCA of murine global lipids from mice on the two diets (**Figure 7**) revealed a much less complex profile of variations than in humans, with the first principal component accounting for 77% of the variance. For this principal component, the higher-fat (Western diet) (WD) samples and the normal (CON) samples were starkly different (Student's t test, p<1e^−6^). The decomposition of the data into linearly independent modes of variability via PCA demonstrated that there are richer modes of variability in human lipid profiles among the normal and diseased states than the variability in WD-fed mice versus control-diet mice, which was rather unidimensional.

## Discussion

Mass spectrometric analysis of phospholipids and diacylglycerols together with computational algorithms can help elucidate biochemical pathways involved in disease progression. This work demonstrates the power and reproducibility of lipidomics techniques in analysis of freshly obtained tissue specimens, particularly from humans. In addition, this analysis shows the important potential for lipidomics to characterize progression of human diseases, in this case NAFLD. Our laboratory has demonstrated the utility of lipidomics profiling in assessing the phospholipid composition of macrophages and their remodeling during zymosan stimulation [10], [16], its use for differentiating the roles of two diacylglycerol kinase (DGK) isoforms in lipid metabolism [17], and in the study of aging and neurodegenerative diseases in a mouse model [18].

This study aimed to characterize the phospholipid and DAG molecular species composition in different stages of NAFLD by MS profiling. Insights into the abundance, paucity, and relative ratios of specific lipid species in this disease could provide insights into signaling pathways relevant in the progression of NAFLD. Lipidomics analysis of tissue extracts has been performed, [19]–[21], but this study is the first to describe systematic analysis of lipid composition in human liver extracts using ESI-MS techniques and to associate the lipidomics results with disease progression. No prior TLC separation by class and derivatization (for GC-MS analysis) is needed. This method is faster and more accurate than previously published and could be used as a high throughput screening technology. This study is complementary to the results from a similar investigation [22] by providing a more detailed account of the fatty acid composition of individual species both in DAG and phospholipids. The method presented herein allowed identification of over 200 individual glycerophospholipid species in each disease state during a single step mass spectrometry analysis. Without further sample manipulation, differences between plasmalogen PC and PE content at each step of the disease were uncovered. Furthermore, the present study has also revealed some important differences in the phospholipid compositions, such as the presence of almost twice as many PS species in human cirrhotic livers compared to normal and steatotic (Table S1). This method was found sensitive enough to allow for the discrimination of differences between disease states for within relatively less abundant lipid classes such as PA, PI, and LPI.

Despite the similar number of glycerophospholipid species detected in all three human sample types, the number of species within each class differed considerably (Table S1). Interestingly, a novel lipid 38∶4 PIe (18:0e/20∶4) was only detected in cirrhotic liver samples. Further investigation of this lipid species may prove it to be a useful biomarker of the disease as well. Another significant observation was the increase of species containing monounsaturated FA (mainly oleate, 18∶1) in the diseased livers when compared to controls (shown in **Figure 2**). This is likely attributable to the excess saturated FA (due to diet or other factors) and probably activation of desaturase enzymes (such as CSD1) [23]. This change in the phospholipid FA composition could possibly have an effect on the triacylglycerol synthesis from DAGs via phosphatidate phosphohydrolase of PA [24].

Comparison of the changes in phospholipid species between murine livers and diseased human livers show several strong similarities. In particular, changes in species of PS and PI from livers of mice maintained on a high fat diet are very similar to the ones observed from human steatotic and cirrhotic specimens. The observed increase in plasmalogen PC and PE in the diseased samples compared to normal could reflect an accumulation in the liver in contrast to the decreased levels of plasmalogen lipids in plasma from NASH and NAFLD cases as a signature of peroxisomal dysfunction [25].

Previous research has focused on the triglyceride accumulation within the liver [26]. Triacylglycerol (TAG), free cholesterol, and cholesterol esters have been reported as elevated as a result of starvation-induced hepatic steatosis [27]. Lipid accumulation in hepatocytes makes them more susceptible to injury by oxidative stress and inflammatory cytokines, whose production in the liver is triggered by the lipid peroxidation products [28], [29]. TAG synthesis (largely regulated by diacylglycerol acyl transferase (DGAT)) protects hepatocytes from free fatty acid (FFA) accumulation [30], thus making its precursor DAG an equally important player.

The most striking changes noted in human and murine samples of steatotic liver specimens were dramatic fold changes in DAG species. A detailed identification of DAG species known to be involved in fundamental intracellular signaling pathways could help to elucidate mechanisms contributing to the progression of NAFLD. Analysis of DAG species demonstrated an increase in saturated and monounsaturated species in steatotic versus normal human specimens, an effect which is more pronounced in the murine data where the magnitude of these species' fold changes was greater (WD/control). Previous reports have connected DAG signaling through protein kinase C (PKC) to processes important in cancer progression [31]–[32]. Changes in specific DAG species such as 38∶4 have been shown to influence PKC activation and consequently regulation of cell proliferation [33]. While this same species of DAG did not demonstrate a large fold change over controls in the steatotic group, many other species demonstrated dramatic fold changes. Among these may be important regulators of proliferation or apoptosis in NAFLD. In addition, specific DAG transferases have been implicated in the progression of NAFLD and liver fibrosis [34], [35]. Modulation of DAG levels as well as their altered fatty acid composition could greatly affect PKC and other pathways, and as such, the progression of NAFLD and perhaps the risk of malignancy in the setting of NAFLD. Previous studies from our group have demonstrated an increase in metastatic tumor burden in the liver in mice with steatotic livers when compared to normal controls [14]. Thus, significant changes in DAGs merit further study to understand their potential roles in tumor initiation and early fibrotic changes in the liver microenvironment.

Not surprisingly, the human liver lipid profiles displayed higher variability than mice maintained on a constrained diet (see error bars in **Figure 2**). The agreement of the DAG changes between human/murine data is substantial, but this is somewhat less true of the glycerophospholipids. To the extent that humans and mice demonstrate strong correlations between the diseased/normal and WD/control lipid differences, in spite of the higher variability in clinical specimens, it is indicative that the model presented here can be used to further study mechanisms of NAFLD progression. In conclusion, this report shows that lipidomics analysis based on MS and computational algorithms is able to assess differences in specific lipid composition in a highly dynamic system such as NAFLD. We have identified upregulated lipid species that can be further explored as potential targets for therapeutic intervention, or which might demonstrate utility as biomarkers relevant to reversible stages of NAFLD. Further work with specific candidate lipids, notably among DAG species, could improve understanding of signaling mechanisms involved in the progression of severe fatty liver disease. In addition, by parallel analysis of murine liver tissues we have shown that mice maintained on a high fat diet can be used as a valuable model to study disease progression, and in the future, to possibly evaluate treatment strategies and outcomes. Based on these novel data, the roles of specific glycerophospholid and DAG species in intracellular signaling, membrane composition, and other vital processes in early and late stage NAFLD can be further examined in this complex disease process.

## Supporting Information

Figure S1 Fragmentation spectra of m/z 871 peak. MS/MS spectra of 38:4e PI from cirrhotic liver sample (eluting at 6.06 min).
(TIF)

Figure S2 MS/MS spectra of 18:0e/20:4 PI synthetic standard. Fragmentation pattern of the liver sample PI yielded fragments consistent with those appearing in the synthetic standard.
(TIF)

Table S1 Total number of identified phospholipid species in each of the human liver (normal, steatotic, and cirrhotic) and murine groups. The number includes the identification of isobaric species (having the same total carbon number and number of double bonds but presented in different fatty acids combinations. I.e., 36:4 PC could be 16:0/20:4, 18:2/18:2, or 18:1/18:3), hence the difference between number of identified phospholipid species and number monitored in the MS scans.
(DOC)
