# Supplementary figures and images for: Increased Diacylglycerols Characterize Hepatic Lipid Changes in Progression of Human Nonalcoholic Fatty Liver Disease; Comparison to a Murine Model
Source: PLoS One. 2011 Aug 9;6(8):e22775. doi: 10.1371/journal.pone.0022775 (PMC3153459; doi:10.1371/journal.pone.0022775)

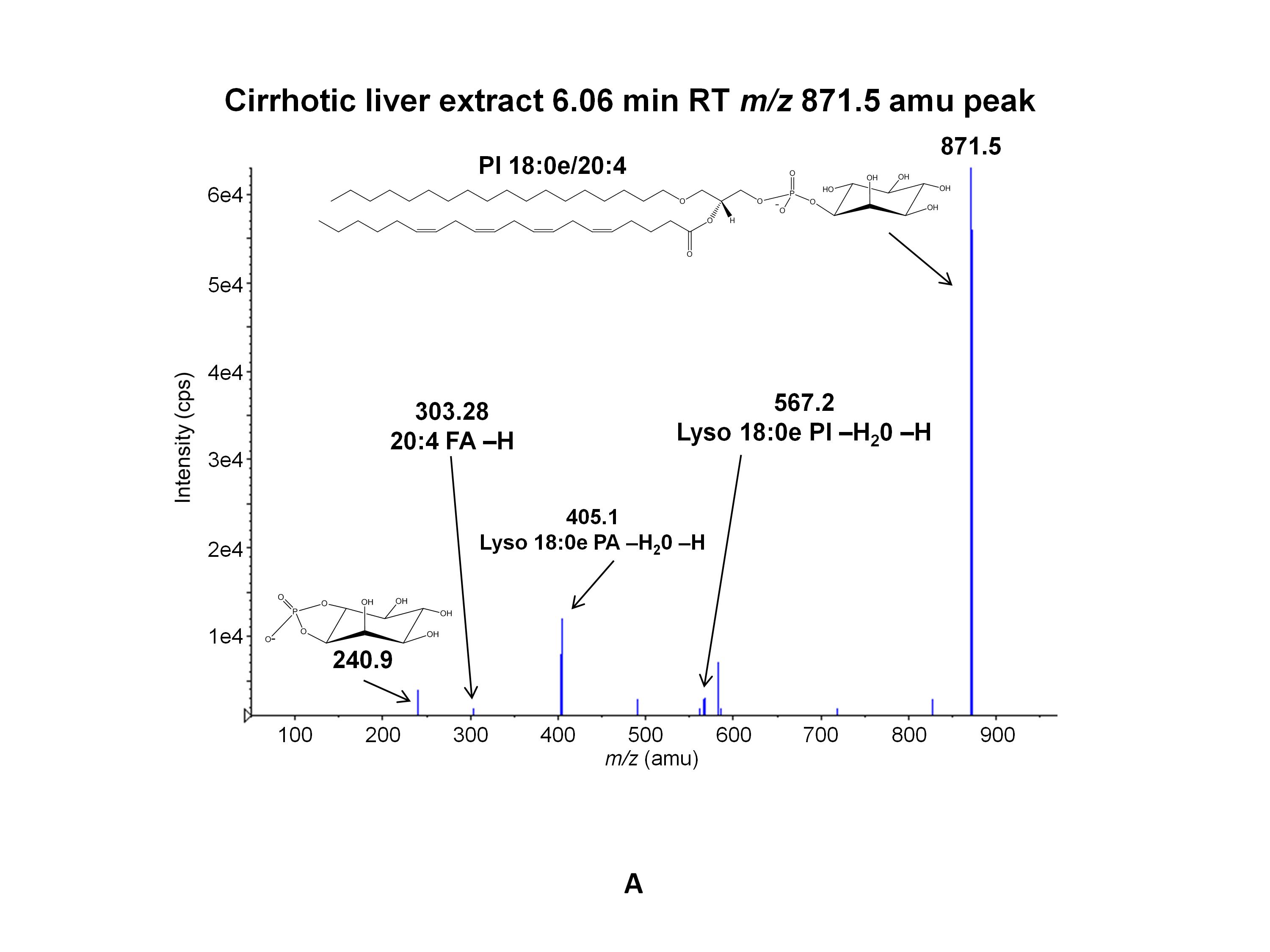

Supplement: Figure S1 — Fragmentation spectra of m/z 871 peak. MS/MS spectra of 38:4e PI from cirrhotic liver sample (eluting at 6.06 min). (TIF) [file pone.0022775.s001.tif]

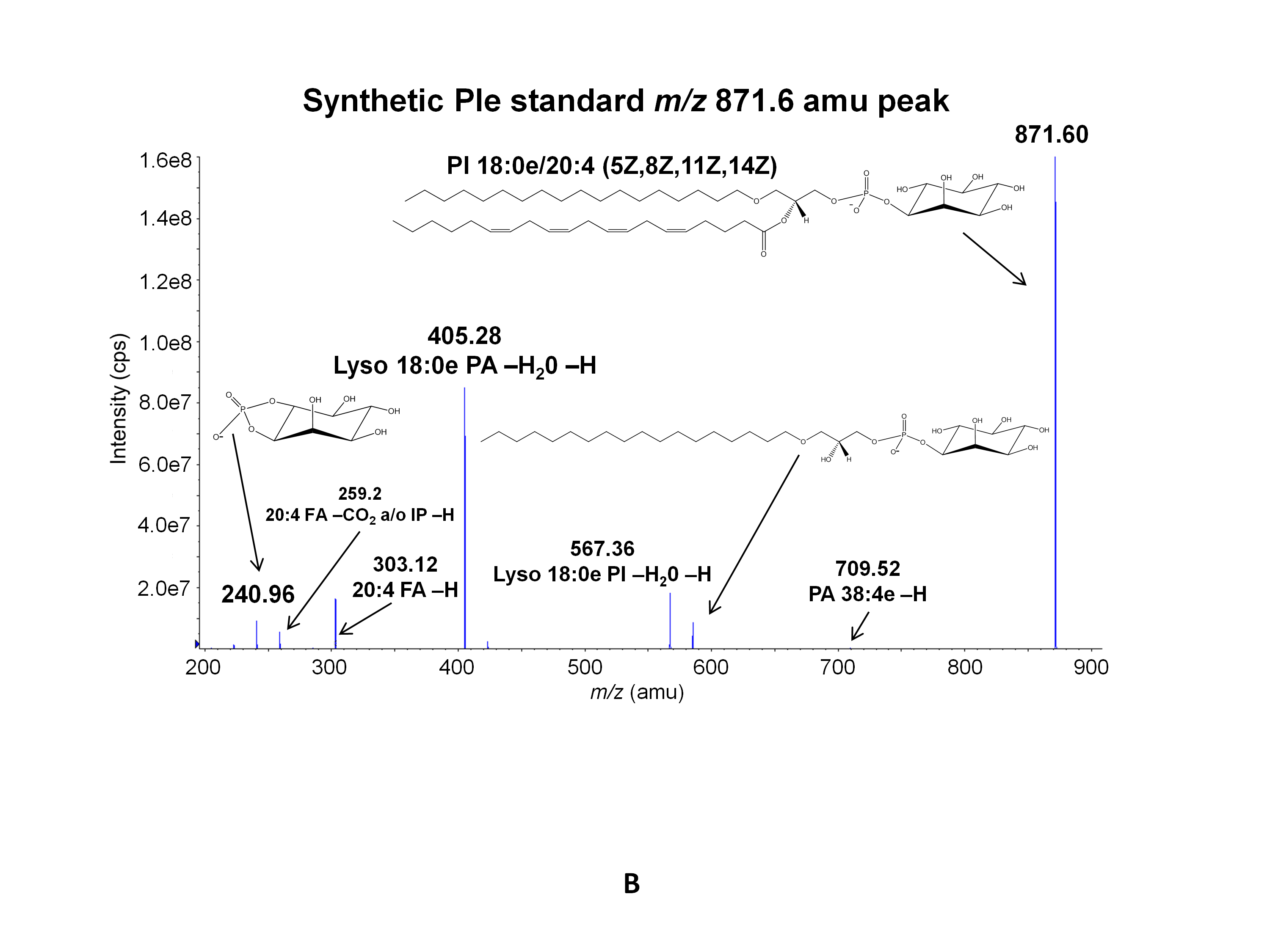

Supplement: Figure S2 — MS/MS spectra of 18:0e/20:4 PI synthetic standard. Fragmentation pattern of the liver sample PI yielded fragments consistent with those appearing in the synthetic standard. (TIF) [file pone.0022775.s002.tif]
