# Supplementary material for: Increased Diacylglycerols Characterize Hepatic Lipid Changes in Progression of Human Nonalcoholic Fatty Liver Disease; Comparison to a Murine Model
Source: PLoS One. 2011 Aug 9;6(8):e22775. doi: 10.1371/journal.pone.0022775 (PMC3153459; doi:10.1371/journal.pone.0022775)
Supplement: Table S1 — Total number of identified phospholipid species in each of the human liver (normal, steatotic, and cirrhotic) and murine groups. The number includes the identification of isobaric species (having the same total carbon number and number of double bonds but presented in different fatty acids combinations. I.e., 36:4 PC could be 16:0/20:4, 18:2/18:2, or 18:1/18:3), hence the difference between number of identified phospholipid species and number monitored in the MS scans. (DOC) [file pone.0022775.s003.doc]

**Supplemental Table 1**. Total number of identified phospholipid species in each of the human liver (normal, steatotic, and cirrhotic) and murine groups. The number includes the identification of isobaric species (having the same total carbon number and number of double bonds but presented in different fatty acids combinations. I.e., 36:4 PC could be 16:0/20:4, 18:2/18:2, or 18:1/18:3), hence the difference between number of identified phospholipid species and number monitored in the MS scans.
